# Paxlovid for Treating COVID-19 Patients: A Case-Control Study From Two Hospitals in the Eastern Province of Saudi Arabia

**DOI:** 10.7759/cureus.39234

**Published:** 2023-05-19

**Authors:** Ali Alsaeed, Abdullah Alkhalaf, Ali Alomran, Walaa Alsfyani, Fadhel Alhaddad, Mousa J Alhaddad

**Affiliations:** 1 Infectious Disease Division, Department of Internal Medicine, Dammam Medical Complex, Dammam, SAU; 2 Department of Internal Medicine, Dammam Medical Complex, Dammam, SAU; 3 Infectious Disease Division, Department of Internal Medicine, Qatif Cental Hospital, Qatif, SAU

**Keywords:** saudi arabia, ritonavir, nirmatrelvir, paxlovid, sars-cov-2, covid-19

## Abstract

Background

Coronavirus disease 2019 (COVID-19) is an infectious disease that shortly progressed into an unprecedented pandemic spreading all over the world and causing millions of deaths. Many new COVID-19-specific therapies were suggested for the treatment of the patients at increased risk of progression to severe disease, especially those who were unvaccinated and those with a likely inadequate vaccine response. One of the preferred therapies in this setting is Paxlovid, a combination of the oral protease inhibitors nirmatrelvir and ritonavir. Paxlovid was authorized by the Saudi Ministry of Health for the treatment of mild to moderate COVID-19. This study aimed to report the effects of Paxlovid on the mortality of the COVID-19 patients at Dammam Medical Complex (DMC) and Qatif Complex Hospital (QCH), two hospitals in the Eastern Provence of Saudi Arabia, and compare the results with the international data.

Methods

The study was a retrospective study that included all the COVID-19 patients who were treated with Paxlovid at DMC and QCH between January and December 2022. Those patients were compared with control COVID-19 patients who did not receive Paxlovid. The patients were included irrespective of their COVID-19 vaccination status. All the patients were managed according to the Saudi Ministry of Health guidelines. They were followed up through the infectious disease virtual clinics and were monitored for ICU admissions and death of any cause for three months following their COVID-19 infections.

Results

A total of 92 COVID-19 patients were included. The patients consisted of 47 male and 45 female patients (51.09% and 48.91%, respectively). The mean ± standard deviation for the patients' age was 55.58±19.25 years. Twenty-eight patients were given Paxlovid (30.43%). Eighteen patients (19.57%) died. The use of Paxlovid was associated with lower ICU admissions (0.0% vs. 18.75%, P value <0.05) and with lower deaths (3.57% vs 26.56%, P value <0.05) but the Paxlovid group included less immunocompromised patients (7.14% vs. 60.94%, P value <0.001), cancer patients (0.0% vs. 42.19%, P value <0.001), and chronic kidney disease patients (7.14% vs. 29.69%, P value <0.05) than the control group.

Conclusion

This study suggests that Paxlovid is highly effective in reducing the risk of severe COVID-19 or mortality. However, larger studies with better qualities are needed for a full assessment of the role of Paxlovid in COVID-19 management in Saudi Arabia.

## Introduction

Coronavirus disease 2019 (COVID-19) is an infectious disease caused by severe acute respiratory syndrome coronavirus 2 (SARS-CoV-2) and was first identified in December 2019 in Wuhan, Hubei Province, China [1]. This disease manifests with respiratory symptoms that could progress to respiratory failure and death in the severely infected patients [2]. It shortly progressed to an unprecedented pandemic spreading all over the world and causing millions of deaths [3].

COVID-19 vaccines were the most promising approach to control the COVID-19 pandemic as they were associated with a mortality reduction, but some individuals could not be fully vaccinated because they developed severe allergic reactions (e.g., anaphylaxis) to a previous COVID-19 vaccine dose [3-6]. Others refused to take the COVID-19 vaccines due to the growing vaccine hesitancy [7]. Even for the vaccinated patients, the protective effectiveness of the COVID-19 vaccines was limited as a suboptimal response was observed in certain populations (e.g., elderly patients and renal cell transplant recipients and other immunocompromised patients) [5,8]. There were also reports of waning response over time [9,10]. Thus, many new COVID-19-specific therapies were suggested for the treatment of patients at an increased risk of progression to severe disease, especially those who were unvaccinated and those with a likely inadequate vaccine response. One of the preferred therapies in this setting is Paxlovid that is a combination of the oral protease inhibitors nirmatrelvir and ritonavir. Nirmatrelvir inhibits SARS-CoV-2 viral replication by blocking its main protease [11]. On the other hand, ritonavir, a human immunodeficiency virus type 1 (HIV-1) protease inhibitor, has no activity against the SARS-CoV-2 virus, but it slows the metabolism of nirmatrelvir and boosts its concentration in the body for longer periods [12].

The combination of nirmatrelvir plus ritonavir could reduce the risk of COVID-19-associated hospitalization and death [13-15]. It received its first emergency use authorization in the United States of America (USA) and its first conditional authorization in the United Kingdom (UK) in December 2021 for the treatment of COVID-19 patients at increased risk of progression to severe disease [12]. Similarly, the Saudi Ministry of Health added Paxlovid to its COVID-19 therapeutic protocol on April 4, 2023, as a recommended option for all the mildly to moderately symptomatic COVID-19 patients older than 50 years of age regardless of risk factors and for the younger adults, if they have one or more risk factors for disease progression [16].

This study aimed to report the effects of Paxlovid on the morbidity and mortality of the COVID-19 patients at Dammam Medical Complex (DMC) and Qatif Complex Hospital (QCH), two hospitals in the Eastern Provence of Saudi Arabia.

## Materials and methods

This was a retrospective study that included COVID-19 patients who were treated with Paxlovid at DMC and QCH between January and December 2022. The Paxlovid group patients were compared with control COVID-19 patients who did not receive Paxlovid, based on the patients' demographic data (e.g., age and gender), comorbid conditions (e.g., diabetes mellitus and hypertension), vaccination status, initial laboratory results, use of steroids, and outcomes (ICU admissions and death). Patients under the age of 18 years were excluded from this study. All the patients were managed according to the Saudi Ministry of Health guidelines [16]. They were followed up through the infectious disease virtual clinics and were monitored for ICU admissions and death of any cause for three months following their COVID-19 infections. The study was approved and monitored by the Institutional Review Board (IRB) of DMC (INF-04, January 29, 2023).

Cancer patients on active chemotherapy, patients with haematological malignancies, solid organ transplant recipients, advanced HIV patients and patients receiving high-dose corticosteroids, antimetabolites, calcineurin inhibitors and biologic agents were labelled as "immunocompromised patients".

The data were analysed using the Python programming language, version 3.7.6 (Python Software Foundation, Wilmington, DE) with the use of the SciPy library 1.4.1 (Enthought, Inc., Austin, TX), and statsmodels module (v0.11.1, Python package). Descriptive statistics (i.e., mean, standard deviation, count, and percentage) were calculated as necessary. Categorical variables were compared with the chi-square test, and continuous variables were compared with the two-sample t-test. A P-value of less than 0.05 was assumed to indicate statistical significance.

## Results

A total of 92 COVID-19 patients were included. The patients consisted of 47 male and 45 female patients (51.09% and 48.91%, respectively), with a male-to-female ratio of 1.04. Most of the patients were Saudis (78 patients, 84.78%). The mean ± standard deviation for the patients' age was 55.58±19.25 years. Fifty-four patients (58.7%) were hypertensive. Forty-six patients (50.0%) were diabetic. Forty-one patients (44.57%) were immunocompromised including 27 cancer patients (29.35%). Eighteen patients (19.57%) were not known to have any chronic medical illness. Forty-four patients (47.82%) had one to two comorbid conditions. The remaining 30 patients (32.61%) had three or more comorbid conditions. Most of the patients received two (39 patients, 42.39%) or three (26 patients, 28.26%) COVID-19 vaccine doses. Patients' demographics are shown in Table 1.

**Table 1:** Patients' demographics (n = 92)

Characteristic		n (%)
Age (mean ± SD, years)		55.58±19.25
Gender	Male	47 (51.09%)
	Female	45 (48.91%)
Nationality	Saudi	78 (84.78%)
	Non-Saudi	14 (15.22%)
Hypertension		54 (58.7%)
Diabetes mellitus		46 (50.0%)
Immunocompromised		41 (44.57%)
Cancer		27 (29.35%)
Chronic kidney disease		21 (22.83%)
Lung diseases		19 (20.65%)
Ischemic heart disease		15 (16.3%)
Sickle cell disease		2 (2.17%)
COVID-19 vaccine doses	0	16 (17.39%)
	1	10 (10.87%)
	2	39 (42.39%)
	3	26 (28.26%)
	4	1 (1.09%)

Patients' initial white blood cells, neutrophils and lymphocytes were 7.78±5.48, 5.75±5.13, and 1.34±1.31 ×10^9^/L, respectively, with a neutrophil-to-lymphocyte ratio of 6.86±8.55. The means ± standard deviations for patients' initial creatinine and albumin were 1.53±1.9 mg/dL and 3.27±0.81 g/L, respectively. Patients' initial laboratory results are shown in Table 2.

**Table 2:** Patients' initial laboratory results (n = 92)

Characteristic	Mean ± SD	Normal range
White blood cells	7.78±5.48	4-10 ×10^9^/L
Neutrophils	5.75±5.13	2-7.5 ×10^9^/L
Lymphocytes	1.34±1.31	1.5-4 ×10^9^/L
Hemoglobin	11.4±2.52	11.5-15.5 g/dL
Platelets	239.03±117.09	150-450 ×10^9^/L
Creatinine	1.53±1.9	0.5-0.9 mg/dL
Albumin	3.27±0.81	3.2-4.8 g/L

Thirty-six patients received steroids (39.13%). Twenty-eight patients were given Paxlovid (30.43%); 13 patients needed ICU admission (14.13%). Eighteen patients (19.57%) died.

The use of Paxlovid was associated with lower ICU admissions (0.0% vs. 18.75%, P value <0.05) and deaths (3.57% vs. 26.56%, P value <0.05), but the Paxlovid group included less immunocompromised patients (7.14% vs. 60.94%, P value <0.001), cancer patients (0.0% vs. 42.19%, P value <0.001), chronic kidney disease patients (7.14% vs. 29.69%, P value <0.05) and less number of comorbid conditions per patient (1.14±1.18 vs. 2.08±1.2, P value <0.001) than the control group. They also had higher hemoglobin (12.93±2.42 vs. 10.81±2.32 g/dL, P value <0.001) and albumin (3.91±0.72 vs. 3.0±0.69 g/L, P value <0.001) levels compared with the control group. A detailed comparison between the COVID-19 patients in the Paxlovid group and the control group is shown in Table 3.

**Table 3:** Comparison between the COVID-19 patients in the Paxlovid group and the control group (n = 92) *A P value less than 0.05 was used to indicate statistical significance.

Characteristic	Paxlovid group (n = 28)	Control group (n = 64)	P value
Age, mean ± SD (years)	55.29±22.27	55.7±17.97	0.924
Male sex, count (%)	15 (53.57%)	32 (50.0%)	0.929
Hypertension, count (%)	14 (50.0%)	50 (62.5%)	0.373
Diabetes mellitus, count (%)	12 (42.86%)	34 (53.12%)	0.497
Immunocompromised, count (%)	2 (7.14%)	39 (60.94%)	0.0000*
Cancer, count (%)	0 (0.0%)	27 (42.19%)	0.0001*
Chronic kidney disease, count (%)	2 (7.14%)	19 (29.69%)	0.0357*
Lung disease, count (%)	4 (14.29%)	15 (23.44%)	0.473
Comorbid conditions per patient, mean ± SD	1.14±1.18	2.08±1.2	0.0008*
COVID-19 vaccine doses, mean ± SD	2.07±1.3	1.75±0.93	0.1814
Initial white blood cells, mean ± SD (×10^9^/L)	8.1±3.24	7.65±6.15	0.735
Initial neutrophils, mean ± SD (×10^9^/L)	5.85±3.23	5.71±5.73	0.907
Initial lymphocytes, mean ± SD (×10^9^/L)	1.36±0.83	1.33±1.46	0.934
Initial neutrophil-to-lymphocyte ratio	6.59±5.45	6.96±9.52	0.857
Initial hemoglobin, mean ± SD (g/dL)	12.93±2.42	10.81±2.32	0.0003*
Initial platelets, mean ± SD (×10^9^/L)	244.38±118.63	236.96±117.39	0.794
Initial creatinine, mean ± SD (mg/dL)	1.03±0.82	1.72±2.16	0.133
Initial albumin, mean ± SD (g/L)	3.91±0.72	3.0±0.69	0.0000*
Use of steroids, count (%)	5 (17.86%)	31 (48.44%)	0.011
ICU admission, count (%)	0 (0.0%)	12 (18.75%)	0.0339*
Death, count (%)	1 (3.57%)	17 (26.56%)	0.0231*

## Discussion

A lot of therapies were thought to be effective against SARS-CoV-2 but failed later to demonstrate a positive effect in well-designed clinical trials. Examples of these agents include hydroxychloroquine, azithromycin, ivermectin, fluvoxamine and metformin [17-21]. Paxlovid, on the other hand, is an oral antiviral therapy consisting of a combination of nirmatrelvir and ritonavir that received an emergency use authorization in the USA and a conditional authorization in the UK [12].

Our results showed that Paxlovid was associated with decreased risks of ICU admission and death. The results were in line with the previously published studies that supported the use of Paxlovid as a treatment for COVID-19 patients at risk of disease progression [13-15,22]. However, they should be interrupted with caution as the control group contained more immunocompromised patients, more patients with comorbid conditions (e.g., cancer and chronic kidney disease), and more anemic patients. They also could have been sicker than the Paxlovid group as evident by the lower values of albumin, a negative acute phase reactant for systemic inflammation. Therefore, it is expected that the control group will have worse outcomes and higher deaths.

In our experience, the use of Paxlovid was limited by the occasional lack of its availability. Some patients presented late since their symptoms started. Thus, they were not given Paxlovid because they passed the five-day rule for its initiation [16]. However, the strongest barrier for the use of Paxlovid was the drug-drug interactions. Many immunocompromised patients were not given Paxlovid due to the fear of these interactions. For instance, cases of tacrolimus toxicity due the interaction with Paxlovid were reported in solid organ transplant recipients [23-26]. Paxlovid can also cause drug interactions with statins, azole antifungals, warfarin and direct-acting oral anticoagulants (DOACs), which are commonly prescribed to this vulnerable group of patients at increased risk of COVID-19 complications [27]. Moreover, Paxlovid use is contraindicated if administered with many other commonly prescribed medications including the antiplatelet ticagrelor, the antiarrhythmic amiodarone, the antipsychotic clozapine, and the phosphodiesterase-5 enzyme inhibitor sildenafil [28].

Our study has several limitations that should be considered. The retrospective nature of the study, the relatively small sample size and the unbalanced groups had greatly affected the generalizability of our results. Studies with better qualities are needed to overcome these limitations and draw final conclusions.

## Conclusions

In keeping with the current literature, Paxlovid use in COVID-19 patients was shown in our study to be associated with a lower risk of death. However, given the nature of the study and the presence of limitations, larger well-controlled clinical trials are needed for a full assessment of the role of Paxlovid in COVID-19 management in Saudi Arabia.
